# PHGDH-mediated serine synthesis in astrocytes supports neuroinflammation by sustaining NADH level to promote histone acetylation

**DOI:** 10.1038/s41419-025-07732-8

**Published:** 2025-05-18

**Authors:** Mengfei Lv, Zhongying Duan, Jinhua Tan, Jiake Liu, Qinqin Wang, Congxiao Wang, Zhaolong Zhang, Xiaona Sun, Rui Liu, Yu Cui

**Affiliations:** 1 Institute of Neuroregeneration and Neurorehabilitation, Qingdao University, Qingdao, Shandong China; 2 Qingdao Medical College, Qingdao University, Qingdao, China; 3 Shandong Collaborative Innovation Center for Diagnosis, Treatment and Behavioral Interventions of Mental Disorders, Institute of Mental Health, Jining Medical University, Jining, Shandong China; 4 Department of Interventional Radiology, The Affiliated Hospital of Qingdao University, Qingdao, Shandong China

**Keywords:** Parkinson's disease, Astrocyte

## Abstract

Neuroinflammation contributes to the loss of dopamine neurons and motor dysfunctions in Parkinson’s disease (PD). How cell metabolism regulates neuroinflammation by modulating epigenetic modifications is largely unknown. In this study, we found that the expression of phosphoglycerate dehydrogenase (PHGDH) which catalyzes the first step of the de novo serine synthesis pathway was mainly expressed in astrocytes and l-methyl-4-phenyl-l,2,3,6-tetrahydropyridine (MPTP) injection triggered the upregulation of PHGDH in astrocytes in substantia nigra. PHGDH inhibition or knockdown reduced proinflammatory cytokine production in primary astrocytes after LPS (lipopolysaccharide) stimulation which was not due to suppressed inflammatory signaling transduction. Mechanistically, PHGDH promotes proinflammatory cytokine transcription by sustaining nicotinamide adenine dinucleotide (NADH) accumulation to facilitate histone acetylation of cytokine promoters. Moreover, PHGDH inhibition-induced inflammatory response decreased neurotoxicity in vitro and alleviated astrocytes-mediated neuroinflammation and neurotoxicity in an MPTP mice model. This study reveals the role and mechanism of PHGDH-mediated serine synthesis in promoting the inflammatory response of astrocytes which may provide a potential target for neurological diseases involving neuroinflammation.

## Introduction

Parkinson’s disease (PD) is the second most common neurodegenerative disease besides Alzheimer’s disease. Protein aggregation, mitochondrial dysfunction, oxidative stress and neuroinflammation have been considered as the main factors that contribute to the loss of dopamine neurons and motor dysfunctions in PD [1]. During neurodegeneration, brain microglia and astrocytes respond to stimuli from injured neurons by altering cellular morphology and producing various kinds of inflammatory cytokines to exacerbate the progression of PD [2, 3]. Thus, elucidating the molecular mechanism underlying neuroinflammation is essential for the therapy and drug development for PD.

Serine is a nonessential amino acid that can either be taken up from the extracellular microenvironment or synthesized de novo in the cytoplasm [4]. Serine exerts important functions in brain development and pathological conditions [5, 6]. L-serine is neuroprotective by promoting the survival and differentiation of neural stem cells (NSCs) [7, 8] and enhancing neuronal synaptic activity [9, 10]. The role of serine in inflammation is diverse. Some studies showed that serine sustains macrophage IL-1β production [11–13]. Other studies found that serine metabolism orchestrates macrophage polarization [14] and antagonizes macrophage antiviral immune response [4]. In the brain, L-serine injection has been revealed to reduce microglia-mediated neuroinflammation after traumatic brain injury [15, 16]. The role of in vivo-synthesized serine in astrocytes-mediated neuroinflammatory response remains unknown.

Phosphoglycerate dehydrogenase (PHGDH) catalyzes the first step of the de novo L-serine synthesis pathway by catalyzing the oxidation of 3-phosphoglycerate (3-PG) derived from glycolysis to 3-phosphohydroxypyruvate (3-PHP) by NAD^+^-coupled redox reactions. PHGDH-mediated serine synthesis has important functions in both physiology and pathological situations by participating in one-carbon metabolism to generate various metabolites such as nucleotides, glutathione (GSH) and S-adenosylmethionine (SAM) [17, 18]. PHGDH regulates bone development and muscle development [19–21]. In addition, PHGDH also participates in tumor progression [22, 23], metabolic diseases [24, 25] and immune-related diseases [4, 14]. The role of PHGDH in neuroinflammation and PD is poorly understood.

In this study, we found that PHGDH was specifically expressed in astrocytes and MPTP injection triggered its upregulation. Inhibition of PHGDH reduces inflammatory cytokine production in astrocytes after LPS stimulation. Mechanistically, PHGDH inhibition led to reduced NADH to decrease histone H3K9/27 acetylation, which finally suppressed transcription of proinflammatory cytokines. Moreover, PHGDH inhibition alleviated astrocytes-mediated neuroinflammation and reduced neurotoxicity in a l-methyl-4-phenyl-l,2,3,6-tetrahydropyridine (MPTP) model.

## Results

### PHGDH is mainly expressed in astrocytes and MPTP injection triggers its upregulation

**Fig. 1:**
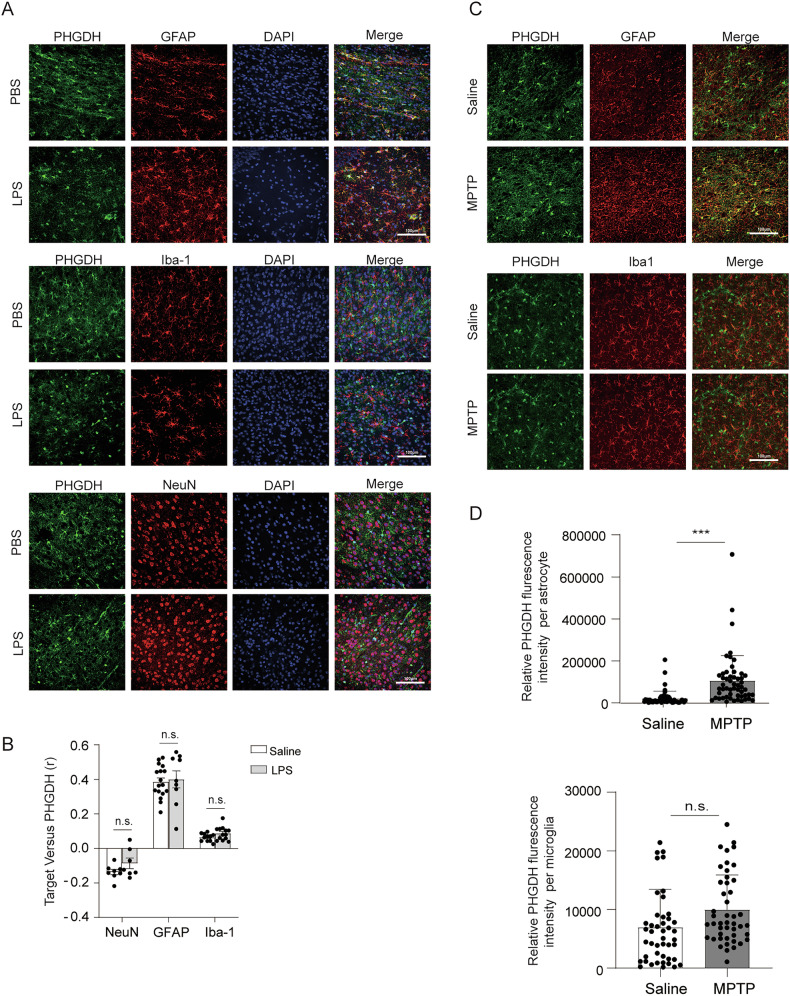
PHGDH is mainly expressed in astrocytes and MPTP triggers its upregulation. **A**, **B** The immunofluorescence staining of PHGDH, GFAP, Iba-1, NeuN and DAPI in brain tissue of C57BL/6 mice before and after LPS stimulation and the statistics of colocalization calculated as Pearson’s correlation coefficient, r. Scale, 100 μm, *n* = 7. **C** immunofluorescence staining representation of PHGDH, GFAP and Iba-1 in substantia nigra of brain tissue of C57BL/6 mice induced by MPTP. Scale, 100 μm, *n* = 4. **D** Statistics of relative PHGDH fluorescence intensity per astrocyte. 54 cells from 6 mice. Statistics of relative PHGDH fluorescence intensity per microglia. 45 cells from 4 mice. The data are means ± SD, for all panels: *P < 0.05, **P < 0.01, ***P < 0.001, n.s. no significance by two-way ANOVA analysis followed by Bonferroni Test (**B**) and one-way ANOVA analysis followed by Student’s *t*-test (**D**). All data are representative of or combined from at least three independent experiments.

To investigate whether PHGDH is involved in astrocyte-mediated inflammation, we first examined its expression. Notably, PHGDH was mainly expressed in astrocytes and weakly expressed in neurons and microglia in brains from C57BL/6 mice under physiological conditions (Fig. 1A). The expression of PHGDH in astrocytes or other brain cells was comparable in brains from saline or LPS-injected mice (Fig. 1B). Notably, the expression of PHGDH in astrocytes was significantly increased after MPTP injection in the substantia nigra (SN) (Fig. 1C, D). No significant expression of PHGDH was observed in microglia after injection of MPTP (Fig. 1D). These results indicated that PHGDH was mainly expressed in astrocytes, and MPTP injection triggered its upregulation.

### PHGDH inhibition or knockdown reduces the expression of proinflammatory cytokine in astrocytes

**Fig. 2:**
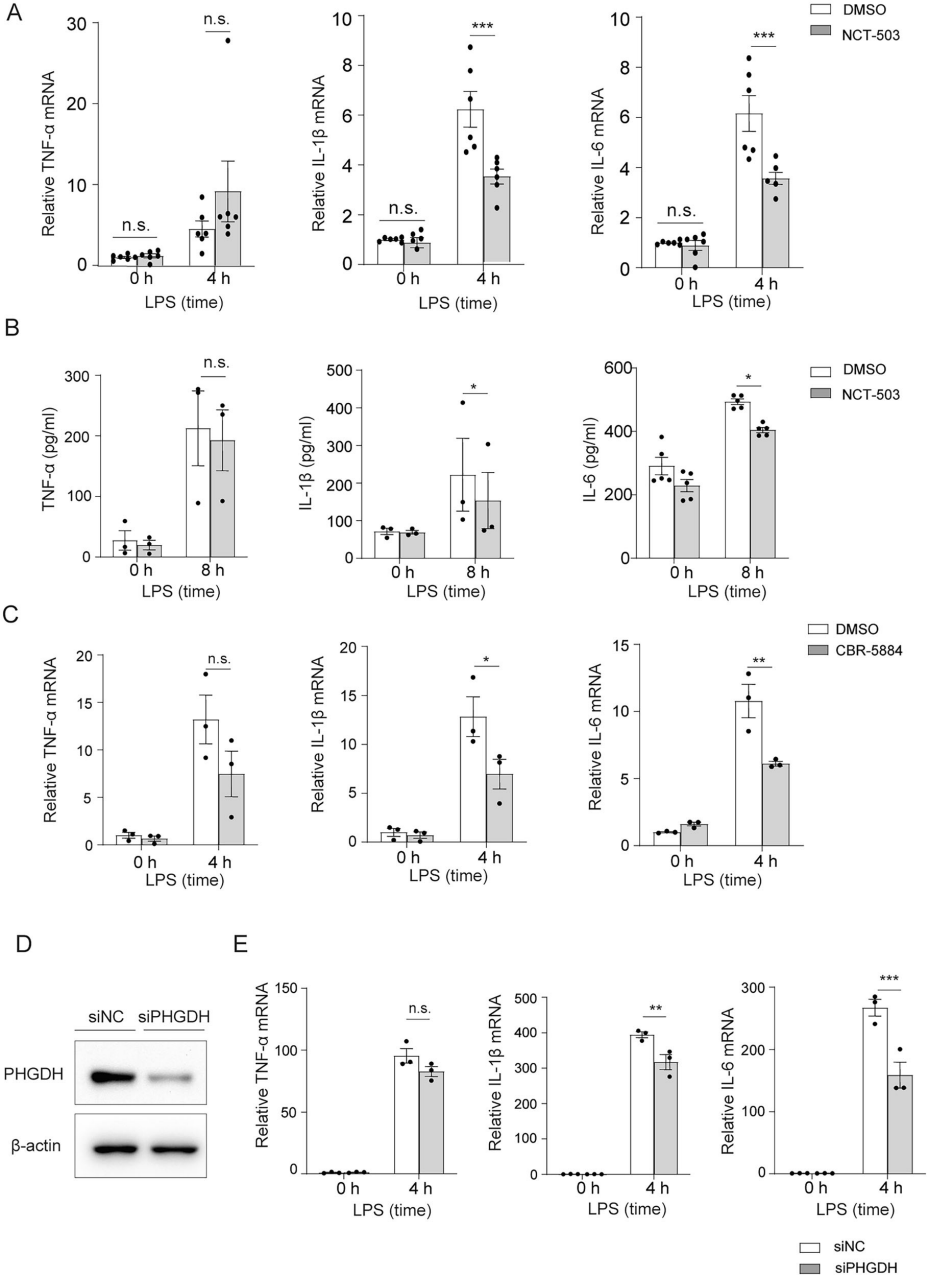
PHGDH inhibition or knockdown reduces the expression of proinflammatory cytokine in astrocytes. **A** qRT-PCR analysis of TNF-α, IL-1β, and IL-6 mRNA expression in astrocytes treated with CNT-503 or DMSO followed by LPS stimulation for 4 h, *n* = 6. **B** ELISAs analysis of TNF-α, IL-1β and IL-6 levels in the supernatants from the cultures of astrocyte-treated with DMSO or NCT-503 followed by LPS treatment of LPS for 8 h, *n* = 3. **C** qRT-PCR analysis of TNF-α, IL-1β and IL-6 mRNA expression in astrocytes treated with CBR-5884 or DMSO followed by treatment with LPS for 4 h, *n* = 3. **D** Representative immunoblotting of siPHGDH or siNC transfected astrocytes. **E** qRT-PCR analysis of TNF-α, IL-1β, and IL-6 mRNA expression in siPHGDH or siNC transfected astrocytes followed by LPS stimulation for 4 h, *n* = 3. The data are means ± SD, for all panels: *P < 0.05, **P < 0.01, ***P < 0.001, n.s. no significance by two-way ANOVA analysis followed by Bonferroni Test (**A**–**C**, **E**).

To explore the role of PHGDH in LPS-induced inflammatory response of astrocytes, we first cultured primary astrocytes in vitro and inhibited PHGDH activity with NCT-503, a specific inhibitor of PHGDH. CCK8 results confirmed that NCT-503 did not increase the death of astrocytes (Fig. S1A). We then measured the expression levels of pro-inflammatory cytokines in astrocytes after LPS stimulation. qRT-PCR results showed that inhibition of PHGDH in astrocytes with NCT-503 significantly reduced the mRNA levels of the pro-inflammatory cytokines IL-1β and IL-6 after LPS stimulation compared with control cells, whereas the mRNA levels of TNF-α were not altered (Fig. 2A). Further ELISA results confirmed the reduced secretion of IL-1β and IL-6 in the supernatants of PHGDH-inhibited astrocytes (Fig. 2B). Similarly, another specific inhibitor of PHGDH, CBR-5884, also suppressed the mRNA expression levels of IL-1β and IL-6 in astrocytes, with no alteration of TNF-α (Fig. 2C). Thus, PHGDH inhibition reduced the expression of proinflammatory cytokines IL-1β and IL-6 in astrocytes after LPS stimulation.

To further test whether silencing the expression of PHGDH could also decrease the expression of IL-1β and IL-6, we transfected primary astrocytes with specific siRNA targeting PHGDH, and simulated cells with LPS. Western blot results confirmed the successful knockdown of PHGDH (Fig. 2D). Consistent with PHGDH inhibition, PHGDH silencing also led to reduced expression of IL-1β and IL-6, and comparable expression of TNF-α in astrocytes after LPS stimulation compared with siNC-transfected controls (Fig. 2E). Therefore, both PHGDH inhibition and silencing resulted in reduced expression IL-1β and IL-6 upon LPS stimulation in astrocytes.

### PHGDH fails to affect inflammatory signaling transduction in astrocytes

**Fig. 3:**
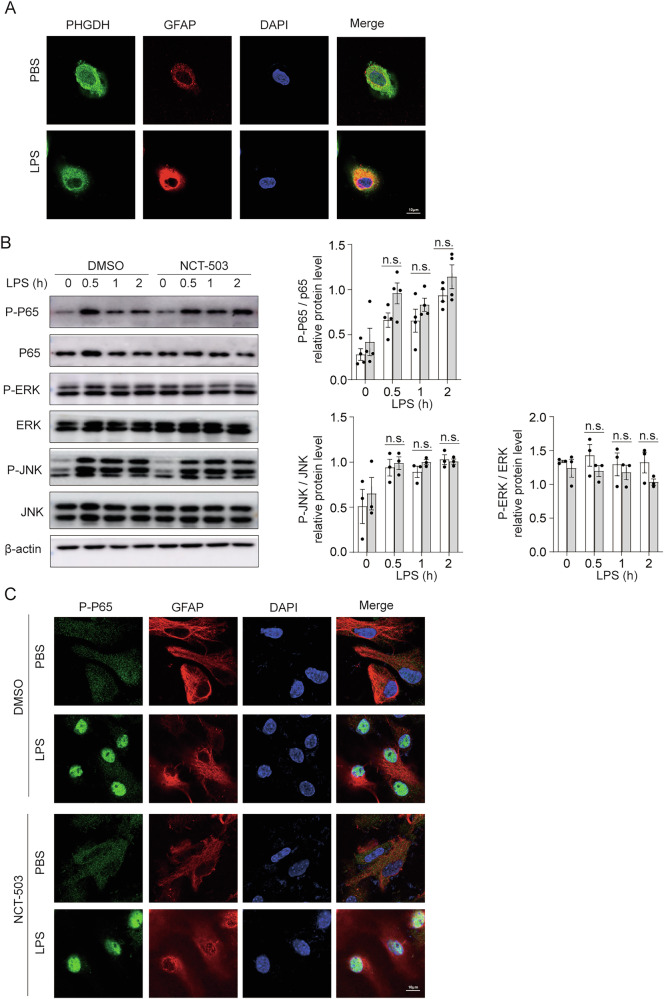
PHGDH fails to affect inflammatory signaling transduction in astrocytes. **A** Representative images of PHGDH, GFAP and DAPI immunofluorescence staining in primary astrocytes treated with LPS or PBS. Scale bar, 20 μm, *n* = 3. **B** Representative immunoblotting of phosphorylation (p-) or total protein of astrocyte lysates treated with DMSO or NCT-503 in the presence or absence of LPS, *n* = 3. **C** Representative images of GFAP, P-P65 and DAPI immunofluorescence staining after astrocytes treated with DMSO or NCT-503 and treated PBS or LPS for 1 h. Scale bar, 10 μm, *n* = 3. The data are means ± SD, for all panels: n.s. no significance by two-way ANOVA analysis followed by Bonferroni Test (**B**).

To identify the potential molecular mechanism by which PHGDH promotes LPS-triggered inflammatory cytokine expression in astrocytes, we first examined the localization of PHGDH in astrocytes before and after LPS stimulation. Cellular immunofluorescence staining showed that PHGDH was mainly localized in the cytoplasm of primary cultured astrocytes either in normal conditions or after LPS stimulation (Fig. 3A). We then examined expression levels of key downstream signaling molecules that are activated by TLR4 signaling, including NFκB, extracellular regulatory protein kinase (ERK), and c-Jun N-terminal kinase (JNK). The activation extent of these signaling pathways was comparable between astrocytes treated with NCT-503 and DMSO (Fig. 3B). These data suggest that the reduced expression of pro-inflammatory cytokines in PHGDH-inhibited cells was not due to compromised activation of pro-inflammatory signals.

It is well known that degradation of IκBα can lead to nuclear translocation of the NF-κB p65 subunit, thereby activating transcription of downstream target genes. We wondered whether PHGDH regulated the translocation of the NF-κB p65 subunit from the cytoplasm to the nucleus. Immunofluorescence results showed that phosphorylated p65 was obviously translocated into the nucleus upon LPS stimulation and NCT-503 treatment did not suppress the translocation of phosphorylated p65(P-p65) from the cytoplasm into the nucleus (Fig. 3C). Taken together, the decreased expression of proinflammatory cytokines in astrocytes owing to PHGDH inhibition did not result from suppressed proinflammatory signal transduction and p65 nuclear translocation.

### PHGDH inhibition limits the production of proinflammatory cytokines through decreased NADH accumulation

**Fig. 4:**
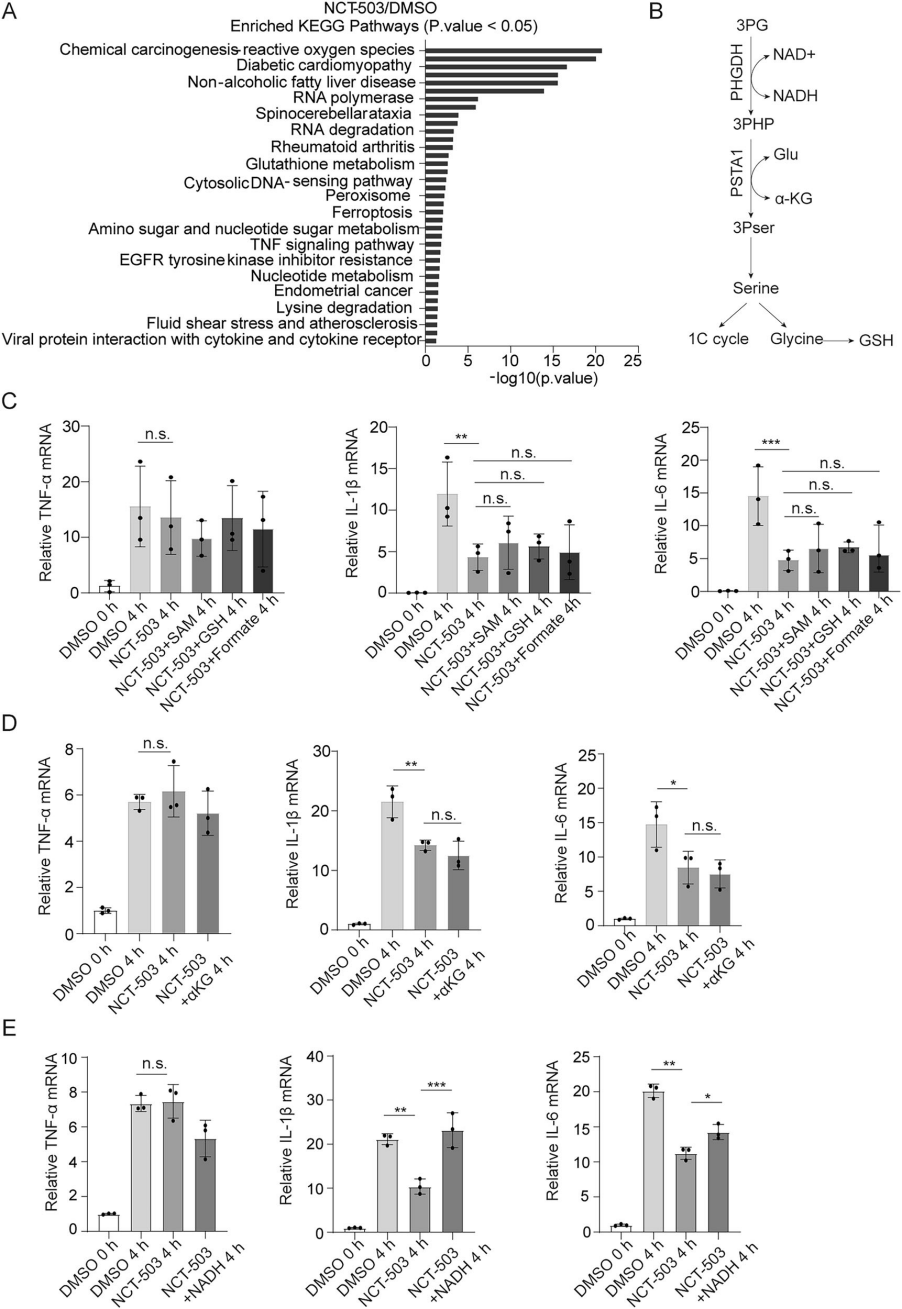
PHGDH inhibition limits the production of proinflammatory cytokines through decreased NADH accumulation. **A** KEGG pathway enrichment analysis of significantly altered genes in primary astrocytes treated with DMSO or CNT-503, based on RNA-seq data, at 4 h after LPS stimulation (*P* < 0.05). **B** Metabolic profile of PHGDH-mediated de novo synthesis of serine in cells. **C** qRT-PCR analysis of TNF-α, IL-1β, IL-6 mRNA expression in astrocytes treated with DMSO or NCT and supplemented with SAM, GSH, or Formate after LPS stimulation for 4 h, *n* = 3. **D** qRT-PCR analysis of TNF-α, IL-1β, IL-6 mRNA expression in astrocytes treated with DMSO or NCT and supplemented with α-KG after LPS stimulation for 4 h, n = 3. **E** qRT-PCR analysis of TNF-α, IL-1β, and IL-6 mRNA expression in astrocytes treated with DMSO or NCT and supplemented with NADH (2 mM) after LPS stimulation for 4 h, *n* = 3. The data are means ± SD, for all panels: *P < 0.05, **P < 0.01, ***P < 0.001, n.s. no significance by one-way ANOVA analysis followed by Tukey’s Multiple Comparison Test (**C**–**E**).

We then performed RNA sequencing (RNA-SEQ) to explore how PHGDH promotes astrocyte-mediated inflammatory cytokine expression. KEGG pathway analysis was conducted to identify the signal pathways enriched for differentially expressed genes (fold change>2; *P* value < 0.05). The pathways associated with downstream serine metabolism, such as nucleotide metabolism and glutathione metabolism were significantly altered (Fig. 4A). We wondered whether downstream metabolites derived from the serine synthesis pathway, such as SAM, GSH or formate were responsible for the modulation of PHGDH on astrocyte-mediated inflammatory response (Fig. 4B). However, the addition of SAM, GSH, or Formate separately failed to rescue the decreased expression of IL-1β and IL-6 in the presence of PHGDH inhibitor NCT-503 (Fig. 4C).

Then we sought to identify whether the metabolites involved in the process of de novo serine synthesis and regulated by PHGDH inhibition including α-KG and NAD+ contributed to the suppressed expression of IL-1β and IL-6. Notably, α-KG supplementation also failed to rescue the expression IL-1β and IL-6 after PHGDH inhibition (Fig. 4D). However, the addition of NADH could completely block the influence of PHGDH inhibition on the expression of inflammatory cytokines IL-1β and IL-6 (Fig. 4E). Taken together, PHGDH promoted the production of proinflammatory cytokines through intracellular accumulation of NADH.

### PHGDH sustains NADH level to increase histone acetylation of cytokine promoters

**Fig. 5:**
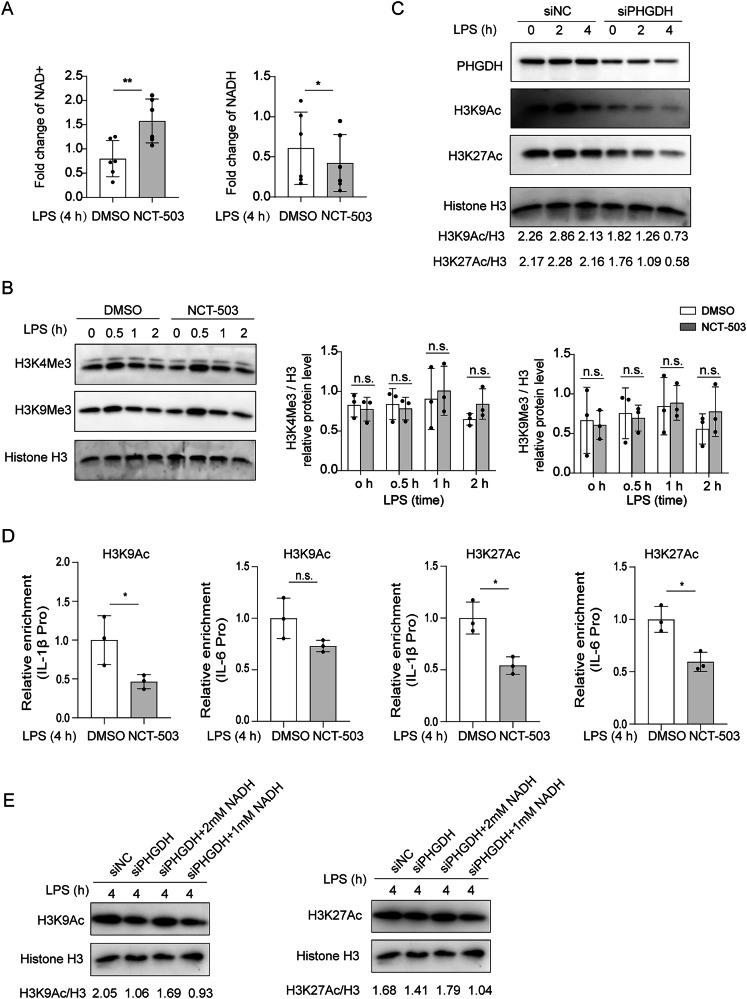
PHGDH sustains proinflammatory cytokine production through NADH-histone acetylation axis. **A** The relative abundance of NAD^+^ and NADH in astrocytes after LPS treatment in the presence of DMSO or NCT-503. **B** Representative immunoblot images and analysis of H3K4me3 and H3K9me3 of DMSO or NCT-503-treated astrocytes after LPS stimulation for 0, 0.5, 1 and 2 h, *n* = 3. **C** Immunoblot representative images of H3K9Ac and H3K27Ac of siNC or siPHGDH-transfected astrocytes after LPS stimulation for 0, 2, and 4 h, *n* = 3. **D** Relative enrichment of H3K9Ac or H3K27Ac in the gene promoter regions of IL-1β and IL-6, *n* = 3. **E** Representative immunoblot images of H3K9Ac and H3K27Ac of siNC or siPHGDH-transfected astrocytes in the presence or absence of 1 mM and 2 mM NADH after LPS stimulation for 4 h. (The loading control was duplicated for detecting H3K9Ac and H3K27Ac, see Supplementary Materials for original WB blots), *n* = 3. The data are means ± SD, for all panels: *P < 0.05, **P < 0.01, ***P < 0.001, n.s. no significance by Student’s *t* test (**A**, **D**) and two-way ANOVA analysis followed by Bonferroni Test (**B**).

As the cytoplasmic transduction of inflammatory signaling and the translocation of P-p65 was not altered after PHGDH inhibition and NAD+ is a well-established Sirtuins (SIRTs) cofactor to regulate histone acetylation, we wondered whether PHGDH-mediated NADH accumulation could regulate histone modifications. The abundance of NAD+ was increased and the level of NADH was decreased after PHGDH inhibition in astrocytes after LPS stimulation (Fig. 5A). Interestingly, PHGDH did not affect H3K4me3 and H3K9me3 in astrocytes after LPS stimulation (Fig. 5B). However, the abundance of H3K9ac and H3K27ac proteins was reduced after PHGDH inhibition and PHGDH silencing in astrocytes (Figs. 5C and S2A, B). In addition, chromatin immunoprecipitation (ChIP) assay showed that PHGDH-inhibition significantly reduced the relative enrichment of H3K9ac and H3K27ac in the regions of regulatory elements of IL-1β and IL-6 in astrocytes (Fig. 5D). Therefore, PHGDH inhibition reduced proinflammatory cytokine production by reducing histone acetylation of IL-6 and IL-1β promoters.

We then investigated how PHGDH inhibition reduced histone acetylation. Previous reports have demonstrated that PHGDH inhibition can lead to the accumulation of NAD+ which may activate SIRTs protein activity to decrease histone acetylation, we first tested whether SIRTs were involved in histone acetylation. Both SIRT1 inhibitor Selisistat (EX 527) and SIRT3 inhibitor 3-triazolylpyridine (3-TYP) failed to rescue IL-6 and IL-1β mRNA levels as well as the abundance of H3K9ac and H3K27ac after PHGDH inhibition suggesting the non-involvement of SIRT proteins in PHGDH-mediated regulation of histone acetylation (Fig. S2C, D). Whereas, supplementation of NADH (2 mM) could significantly recover the reduced abundance of H3K9ac and H3K27ac after inhibiting PHGDH in primary astrocytes (Fig. 5E). Thus, knocking down PHGDH reduced histone acetylation through suppressing NADH levels.

### PHGDH inhibition-induced inflammatory response reduces neurotoxicity in vitro

**Fig. 6:**
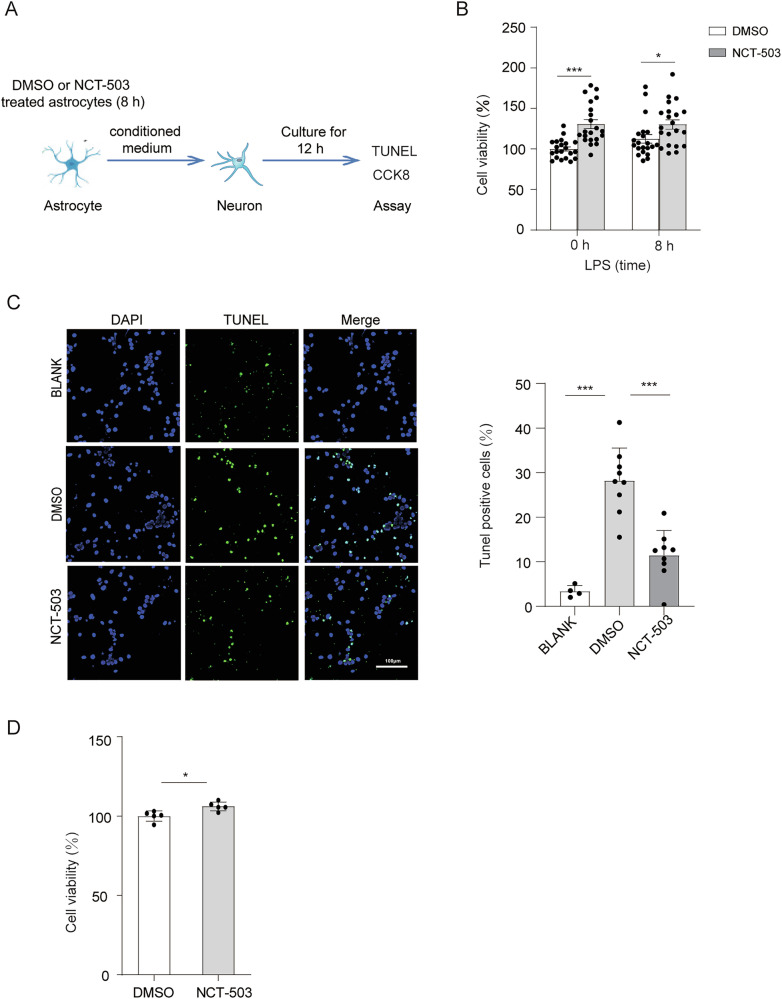
PHGDH inhibition-induced inflammatory response reduces neurotoxicity in vitro. **A** Diagram of co-culture of astrocyte supernatants and neurons. **B** Neuronal viability after co-culturing with astrocyte-conditioned medium measured by CCK8. **C** Representative immunofluorescence staining and quantitative analysis of TUNEL^+^ neurons after co-culturing with astrocyte conditioned medium. Scale, 100 μm, *n* = 3. **D** Neuronal viability in the presence of DMSO or NCT-503. *n* = 5. The data are means ± SD, for all panels: *P < 0.05, **P < 0.01, ***P < 0.001 by two-way ANOVA analysis followed by Bonferroni Test (**B**), one-way ANOVA analysis followed by Tukey’s Multiple Comparison Test (**C**) and Student’s *t*-test (**D**).

Inflammatory cytokines released by activated microglia or astrocytes have been shown to regulate neuronal viability. To investigate whether PHGDH-mediated cytokine expression in astrocytes was preferentially toxic to neurons, we inhibited PHGDH in primary astrocytes and tested the effect of conditioned medium (CM) after LPS stimulation on cell viability of cultured cortical neurons (Fig. 6A). CM from DMSO-treated astrocytes resulted in significant neuronal death, while CM from NCT-503-treated astrocytes had significantly fewer toxic effects on neurons (Fig. 6B). In addition, we also used TUNEL immunofluorescence staining to confirm the effects of the reduced expression of inflammatory cytokines from astrocytes owing to PHGDH inhibition on neuronal viability. Compared with the control group, the frequency of TUNEL^+^ neurons in the NCT-503 treated CMs was significantly reduced (Fig. 6C). To exclude the potential influence of NCT-503 on neurons, we also tested the viability of direct addition of NCT-503 on neuronal viability. CCK8 detection showed that NCT-503 itself had no damage to the survival of neurons (Fig. 6D). Thus, comprised inflammatory response of astrocytes induced by PHGDH inhibition reduced neurotoxicity in vitro.

### Inhibition of PHGDH alleviates neuroinflammation in vivo in MPTP-induced mice

**Fig. 7:**
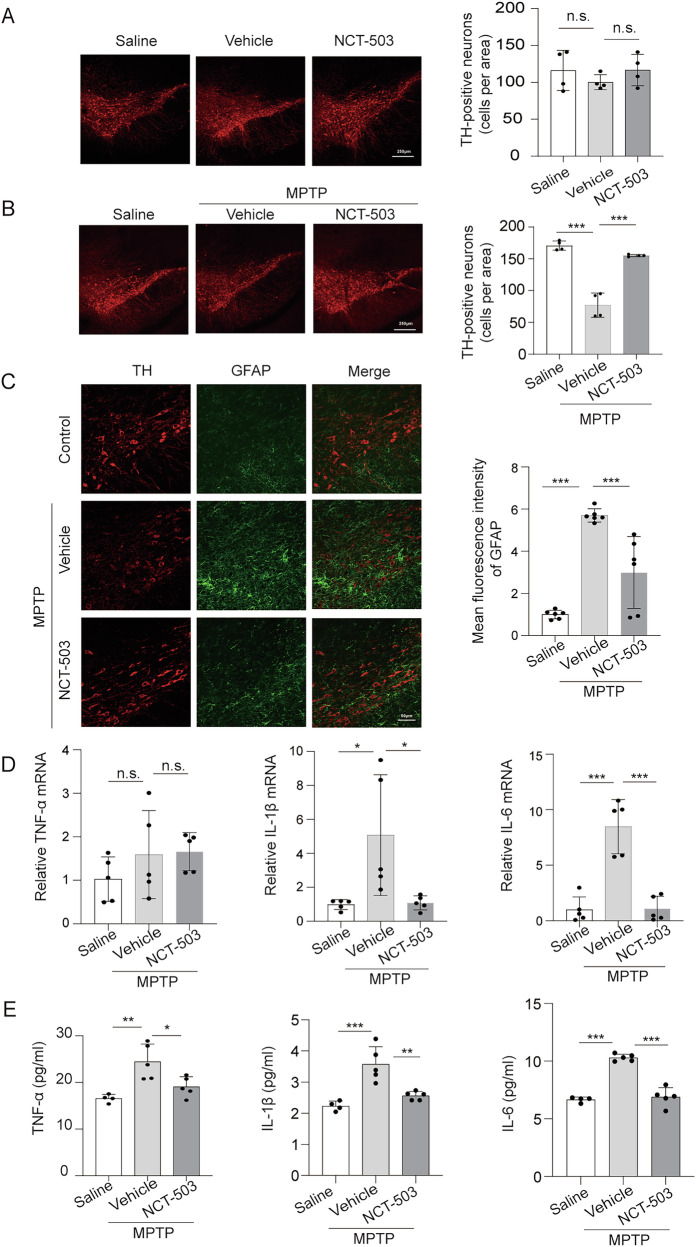
Inhibition of PHGDH alleviates neuroinflammation in vivo in MPTP-injected mice. **A** Representative images of immunofluorescence staining of TH^+^ neurons in the substantia nigra region of C57BL/6 mice treated with saline, vehicle or NCT-503 under physiological conditions. Scale, 250 μm, *n* = 5. **B** Representative immunofluorescence images of TH^+^ neurons in the substantia nigra region of brains after treatment with vehicle or NCT-503 after intraperitoneal injection of MPTP. Scale, 250 μm, *n* = 4. **C** Representative immunofluorescence images and statistical analysis of GFAP intensity in the substantia nigra region of mice brains injected with vehicle or NCT-503 after MPTP injection. Scale, 50 μm, *n* = 6. qRT-PCR analysis (**D**) and (**E**) ELISA analysis of the expression levels of TNF-α, IL-1β and IL-6 in the substantia nigra region of mice treated with vehicle or NCT-503 after MPTP injection, n = 5. The data are means ± SD, for all panels: *P < 0.05, **P < 0.01, ***P < 0.001, n.s. no significance by One-Way ANOVA analysis followed by Tukey’s Multiple Comparison Test (**A**–**E**). All data are representative of three independent experiments.

Previous studies have shown that astrocytes-mediated neuroinflammation is involved in the progression of Parkinson’s disease (PD). To analyze whether PHGDH affected the inflammatory response in mouse models of acute Parkinson’s disease, C57/BL6 mice were intraperitoneally injected with saline or MPTP (20 mg/kg), once every 2 h, for a total of 4 times to induce acute Parkinson’s disease models in mice. Injection of NCT-503 and its dissolving vehicle in normal mice did not affect the survival of tyrosine hydroxylase-positive (TH^+^) neurons (Fig. 7A). We observed obvious loss of TH^+^ neurons in the brain tissue of the substantia nigra of MPTP-injected mice and the death of TH^+^ neurons in the substantia nigra of mice brain tissue decreased after PHGDH inhibition compared with vehicle-injected mice (Figs. 7B and S3A). At the same time, we detected the activation of astrocytes in the substantia nigra region, and immunofluorescence staining results showed that the activation of astrocytes and cell reactivity decreased significantly after PHGDH inhibition (Fig. 7C). Moreover, qRT-PCR results showed the reduced mRNA expression of IL-1β and IL-6 in the substantia nigra region of brain tissue of MPTP-injected model mice with PHGDH inhibition (Fig. 7D). Furthermore, ELISA results confirmed the reduction of IL-1β and IL-6 after PHGDH inhibition, although the abundance of TNFα was also reduced (Fig. 7E). These results demonstrated that PHGDH inhibition could reduce astrocyte-mediated neuroinflammation in an MPTP-induced Parkinson’s disease model.

## Discussion

In this study, we found that PHGDH was mainly expressed in astrocytes and MPTP injection triggered its upregulation. Inhibition of PHGDH reduced inflammatory cytokine production in astrocytes after LPS stimulation. Mechanistically, PHGDH inhibition led to reduced accumulation of NADH which decreased histone H3K9/27 acetylation to suppress cytokine transcription. Moreover, PHGDH inhibition alleviated astrocytes-mediated neuroinflammation and reduced neurotoxicity in a mice MPTP model.

Previous studies showed that the expression level of serine is altered during PD progression. One study showed that D-serine and L-serine levels are increased in the rostral putamen of MPTP-treated monkeys [26]. However, oral supplementation of D-serine did not affect the number of TH^+^ neurons and microglia activation after MPTP injection. Another study showed that D-serine is reduced in the substantia nigra of MPTP-lesioned macaques and in the cerebrospinal fluid of patients suffering Parkinson’s disease [27]. In our study, we found that PHGDH was selectively expressed in astrocytes and MPTP injection upregulated the expression of PHGDH. Notably, inhibition of PHGDH did not directly affect neuronal viability and astrocyte viability but reduced the inflammatory response of astrocytes. In vivo injection of PHGDH inhibitor NCT-503 alleviated MPTP-induced TH^+^ neuronal death which might resulted from reduced neuroinflammatory response of astrocytes. Therefore, serine metabolism participates in PD progression probably by regulating neuroinflammation.

Recently, the role and mechanism of cell metabolism in regulating inflammatory response are being revealed. As an important non-essential amino acid, the role of serine in inflammation has been reported. Some studies showed that serine sustains macrophage IL-1β production by increasing GSH production, mTOR activation or NAD^+^-mediated protein acetylation [11–13]. Wang et al. showed that PHGDH-mediated serine synthesis promotes IL-1β production by increasing TLR4 transcription and NLRP3 acetylation in macrophages. In our study, we found that PHGDH-inhibition reduced IL-1β and IL-6 transcription through NADH-histone acetylation axis and the activation of inflammatory signaling transduction was not observed. Thus, the regulation of PHGDH-mediated serine synthesis in inflammation varies in different immune cells. In addition, another study showed that serine metabolism orchestrates macrophage polarization [14]. As astrocytes can also polarize into proinflammatory or anti-inflammatory phenotypes [28], the role of PHGDH-mediated serine synthesis in astrocyte polarization needs further investigation. Besides the proinflammatory role, several studies also demonstrated the anti-inflammatory role of serine in vivo disease conditions. For example, serine prevents LPS-induced intestinal inflammation and barrier damage via glutathione synthesis and AMPK activation [29]. In the brain, L-serine injection has been reported to reduce microglia-mediated neuroinflammation after traumatic brain injury [16, 17]. As the target cell of L-serine is more complex in vivo, the cause or effect of serine on inflammation should be distinguished in vivo conditions in future studies.

Many previous studies have shown that cell metabolism changes can regulate inflammatory response by reprograming epigenetic modifications [30, 31]. In this study, we demonstrated that PHGDH promoted histone acetylation to facilitate proinflammatory cytokine transcription in a NAD + -independent manner but relied on NADH accumulation. Previous studies showed that NAD+ can regulate histone acetylation by activating SIRT proteins [13, 32]. In addition, NADPH level was also reported to affect cellular epigenetic state by interacting with histone deacetylase 3 (HDAC3) and disrupting the association between HDAC3 and NCOR complex [33]. In this context, it is not surprising that NADH can also affect histone acetylation although the detailed molecular of how NADH specifically regulates histone acetylation still needs further investigation.

In this study, we found that PHGDH was specifically expressed in astrocytes in the brain and hardly expressed in neurons and microglia which is consistent with a previous report [34]. Accordingly, neurons are not able to synthesize L-serine and astrocytes transport serine to support neurons. These data indicate the different sources of serine in different brain cells although the reason for these differences is not understood. Whether other nonessential amino acids also exhibit selective expression in glial cells or neurons is unknown. PHGDH is involved in various physiological and pathological conditions in an enzyme-dependent or independent manner. PHGDH-mediated serine synthesis contributes to one-carbon metabolism and generates various downstream metabolites to participate in tumor growth [22], tissue development [20, 21] or immune-related disease [4]. However, PHGDH can also function in a serine-independent manner. PHGDH can localize to the inner mitochondrial membrane in liver cancer cells and promote mitochondrial translation and respiration to enhance the recycling efficiency of the mitochondrial ribosome [35]. In addition, nutrient deficiency results in the phosphorylation of PHGDH leading to its nuclear translocation which further represses the poly(ADP-ribosyl)ation of c-Jun by PARP1, thereby impairing c-Jun-mediated gene transcription for tumor growth [36]. Currently, whether the selective expression of PHGDH in astrocytes correlates with its nonenzymatic activity is unknown.

In conclusion, our findings indicate that PHGDH-mediated serine de novo synthesis can facilitate astrocyte-mediated neuroinflammation by sustaining NDAH levels to promote histone acetylation of pro-inflammatory cytokines and increase cytokine transcription. Thus, modulating the PHGDH-mediated serine synthesis pathway may serve as a potential therapeutic target for reducing neuroinflammation and improving neuronal repair.

## Methods

### Mice

C57BL/6 mice (9-10-week-old, male and female) were used for MPTP (1-methyl-4-phenyl-1,2,3,6-tetrahydropyridine) model. All experiment animals were purchased from Jinan Pengyue Experimental Animal Breeding Co. LTD and were conducted in compliance with National Institutes of Health guidelines and were approved by the institutional animal care and use committee of Qingdao University (QDU-AEC-2024665).

### Primary astrocytes culture

Primary astrocytes were cultured from the cerebral cortices of 1-2-day-old mice. In brief, meninges and blood vessels were completely removed from the cortices of neonatal brains of C57BL/6 mice and the rest cortex of the brain was dissociated, and were then digested with 0.05% trypsin for 20 min at 37 °C. Cell suspensions were plated in poly-d-lysine-coated (SIGMA, P6407) T75 cell culture flask, and were cultured with DMEM medium supplemented with 10% FBS (Sigma) and 1% antibiotics for 11 days at 37 °C in a humidified incubator with 5% CO_2_. Cell culture media were changed every three days. Microglial cells were removed by shaking the culture flask for 16–24 h at 250 rpm at 37 °C, and the cells that remained attached to the bottom were washed with PBS for three times and digested with 0.25% trypsin for 5 min and harvested for further experiments. The purity of cultured astrocytes was tested to be over 97% based on immunofluorescent staining with GFAP [37].

### Primary cortical neurons culture

Primary cortical neurons were cultured from embryonic day 17 (E17) of C57BL/6 mice embryos. Single-cell suspensions were prepared from the dissected cortices and digested with 0.05% trypsin for 20 min at 37°C, and then the cells were plated at a density of 0.2 × 10^6^ in poly-d-lysine-coated plastic 24-well plates in neurobasal feeding medium. Neurons were cultured in a humidified incubator at 37 °C in 5% CO_2_, culture media were changed every 3 days. After 9 days, neurons were ready for further experiments [38].

### MPTP treatments

Mice subjected to MPTP treatment were given four times intraperitoneal injections of MPTP (20 mg/kg, i.p.) at 2 h intervals, and control mice were received equivalent saline injections [38]. As NCT-503 can penetrate into the brain [39], three injections of NCT-503 (20 mg/kg, i.p.) were administered at 24, 48, and 72 h after MPTP injection. The mice were sacrificed at 7 days after the last injection for following Inflammatory cytokines detection and tissue immunofluorescence staining. The sample size was determined based on previous publications and practical considerations [38, 40]. Power analysis was performed to ensure the study had sufficient statistical power to detect significant effects confidently. To ensure the integrity of the data, a person who was not involved in the experimental procedures was used to allocate the experimental groups of randomization process. Blinding procedures were performed as follows: a researcher who was blinded to the experimental grouping of the animals performed the outcome test.

### siRNA-mediated interference

To silence PHGDH gene expression, 20 nM siRNA was transfected into the primary astrocytes using standard procedures with Lipofectamine RNAiMAX Transfection Reagent according to the manufacturer’s instructions [38]. Astrocytes were stimulated with 100 ng/ml LPS and harvested for RNA or cell extracts for further analysis at 48 h after transfection. The following siRNA sequences were used: mouse PHGDH sense: 5’-UCGGCAGAAUUGGAAGAGAtt-3’; mouse PHGDH anti-sense: 5’- UCUCUUC CAAUUCUGCCGAtt-3’.

### Immunofluorescence, confocal microscopy, and image analysis

Brain tissues were fixed in 4% PFA in phosphate buffer for 12 h at room temperature (RT) and incubated at 4 °C in phosphate buffer containing 30% sucrose for 48 h. The cryosections were permeabilized and blocked in PBS containing 0.3% triton and 10% BSA for 2 h and incubated at 4 °C overnight with the primary antibody against PHGDH (1:1000), GFAP (1:2000, DAKO), Iba1(1:1000, Wako), NeuN (1:1000, Millipore), P-P65(1:1000, CST 3033). After three times washes with PBS, brain sections were stained with secondary antibodies (1:1000) for 1 h. The nuclei were stained with DAPI (S2110, Solarbio). Immunofluorescence intensity was captured using a confocal microscope (Nikon-Ti2-E). Images were analyzed using ImageJ [41].

Primary astrocytes and neurons were fixed with 4% PFA for 10 min and permeabilized with 0.5% Triton-X-100 for 10 min [42]. Subsequently, cells were blocked in 5% BSA in PBS for 2 h RT. Primary antibodies against GFAP, P-P65, PHGDH were incubated overnight at 4 °C and secondary antibodies were incubated at RT for 1 h. Immunofluorescence intensity was captured using a confocal microscope (Nikon-Ti2-E).

### Statistical analysis

GraphPad Prism software was used for statistical analysis. The results of the experiments were shown as mean ± SD. All data were representative or combined from at least three independent experiments. To compare the statistical significance of two independent groups, Student’s *t*-test was used. Differences between the groups were determined using one-way or two-way ANOVA analysis.

## Supplementary information

Supplementary information

Supplementary WB RAW data

## Data Availability

All data generated or analyzed during this study are included in this published article. The RNA-seq data has been deposited in the National Center for Biotechnology Information database under access code PRJNA1153077.
