# Supplementary material for: PHGDH-mediated serine synthesis in astrocytes supports neuroinflammation by sustaining NADH level to promote histone acetylation
Source: Cell Death Dis. 2025 May 18;16(1):397. doi: 10.1038/s41419-025-07732-8 (PMC12086227; doi:10.1038/s41419-025-07732-8)
Supplement: Supplementary file 1 — Supplementary information [file 41419_2025_7732_MOESM1_ESM.pdf]

Supplementary Information

The supplementary information includes Materials and Methods, Supplementary Figures and Figure legends.

Supplementary Materials and Methods

Antibodies and reagents

Antibodies used were listed below: PHGDH Rabbit Ab (14719-1-AP) was purchased from Proteintech. β-actin Abs (66009-1-Ig), HRP-conjugated Affinipure Goat Anti-Mouse IgG (H+L) (SA00001-1) and HRP-conjugated Affinipure Goat Anti-Rabbit IgG (H+L) (SA00001-2) were purchased from Proteintech; Anti-MAP2 antibody (M3696) was from Sigma; Iba-1 (019-19741) was purchased from Wako Chemical; GFAP (Z0334) was purchased from DAKO; NeuN (MAB377) was from Millipore; Phospho-NF-κB p65 (Ser536) (93H1) Rabbit mAb (3033S), NF-kappa-B p65 (C22B4) Rabbit mAb (4764S), Phospho-SAPK/JNK (Thr183/Tyr185) (81E11) Rabbit mAb (4668), SAPK/JNK Antibody (9252), Phospho-p44/42 MAPK (Erk1/2) (Thr202/Tyr204) Antibody (9101S), and p44/42 MAPK (Erk1/2) Antibody (4695) were purchased from Cell Signaling Technology (CST); Donkey anti-mouse IgG-AlexaFluor-488 conjugate (A21202), Donkey anti-mouse IgG-AlexaFluor-568 conjugate (A10037), Donkey-anti-rabbit IgG-AlexaFluor-568 conjugate (A10042) and Donkey-anti-rabbit IgG-AlexaFluor-647 conjugate antibody (A31573) were purchased from Thermo; Chromatin immunoprecipitation (ChIP) Assay Kit (P2080S) was purchased from Beyotime Biotechnology; Acetyl-Histone H3-K9 Rabbit mAb (A21107), Acetyl-

Histone H3-K27 Rabbit mAb (A2771) was purchased from Abclonal; 2-Ketoglutaric acid Sodium (AKG T4920), NCT-503 (T4213) , CBR-5884 (T14884), L-Glutathione reduced (GSH T1085), Cell Counting Kit-8(KA251771), 3-TYP (3-(1H-1,2,3-triazol-4-yl) pyridine) (T4108) and Selisistat (EX-527) (T6111) were purchased from TargetMol; Ademetionine (SAMe S5109) and Ammonium formate (S5382) were purchased from Selleck; NADH/NAD kit (S0175) and One Step TUNEL Apoptosis Assay Kir (Green Flurescence) (C1088) were purchased from Beyotime Biotechnology.

Conditioned medium (CM) assays

Astrocytes cells were incubated with DMSO or NCT (20 μM) for 2 h and then were treated with 0.1 μg/mL LPS (lipopolysaccharide) for 4 h. After that, the supernatants were collected and centrifuged to remove cell debris. Primary cortical neurons were cultured with CMs (diluted with fresh neuron culture medium at a 1:1 ratio) for 12 h to perform TUNEL staining to detect neuronal viability.

CCK8 assay

Cell survival was assayed by Cell Counting Kit-8 (TargetMol) based on the manufacturer's instructions. CCK-8 solution was added into each well, and incubated for indicated times. Cell viability was determined by measuring the OD at 450 nm. Percent over control was calculated as a measurement of cell viability.

Quantitative real-time PCR (qRT-PCR) analysis

Total RNA of brain tissues was isolated using TRIzol reagent (Thermo Fisher Scientific) according to the manufacturer's protocol. Total RNA of cultured cells was extracted

with RNAfast200 purification kit (Fastagen), and reverse-transcribed with the ReverTra Ace® qPCR RT Master Mix with gDNA Remover (TOYOBO) (40). Real-time PCR was performed on the QuantStudio 3 (Thermo Fisher Scientific) with RealStar Green Power Mixture (GenStar). GAPDH was used as the internal control. The sequences of qRT-PCR primers for the genes examined are listed below: GAPDH-Forward Primer (FP): 5'-TGGTGAAGGTCGGTGTGAACGG-3', GAPDH-Reverse Primer (RP):5'-ACTGTGCCGTTGAATTTGCCG-3'; TNFA-FP: 5'-TGATCGGTCCCCAAAGGGATG-3', TNFA-RP: 5'-TTG GTGGTTTGCTACGACGTGG-3'; IL-6-FP: 5'-TGATGC ACTTGCAGAAAACAATCTGA-3', IL-6-RP: 5'-AGCTATGGTACTCCAGAAGAC CAGAGG-3'; IL-1β-FP: 5'-GCAACTGTTCCT GAACTCAACT-3', IL-1β-RP: 5'-AT CTTTTGGGGTCCGTCAACT-3'; PHGDH-FP:5'-CGATGAAAGATGGCAAAT GG -3'; PHGDH-RP: 5'-TGTGGTAGAGGCCAGGAGTG-3'.

Enzyme-linked immunosorbent assay (ELISA)

Secreted cytokines in the supernatants of primary astrocytes from different treatment groups were analyzed using mouse TNFα, IL-6, and IL-1β ELISA kits (Absin Bioscience) according to the manufacturer's instructions.

Immunoblot analysis

Total proteins were extracted from astrocytes or brain tissues with lysis buffer containing protease (1% P6730, Solarbio) and phosphatase inhibitors (1% P1260, Solarbio). The proteins were isolated by SDS-PAGE and transferred to PVDF membranes (Millipore, USA). The membranes were blocked with 5% skim milk or 5% bovine serum albumin (BSA) in Tris-buffered saline-Tween 20 (TBST) for 2 h at RT.

Then the membranes were incubated with primary antibodies overnight and secondary antibodies for 1h. Membranes were exposed with a chemiluminescence detection system (Image Quant 800, Cytiva).

RNA-seq, library generation, and bioinformatic analysis

Primary astrocytes (approximately 1000000 cells) were pre-treated with DMSO or NCT for 2 h, and then stimulated with 0.1 μg/mL LPS for 4 h. RNA was extracted, purified. Eukaryotic referent transcriptome sequencing (RNA-seq) uses Illumina sequencing platform to sequence all mrnas transcribed in a specific tissue or cell in a specific state of eukaryotes, align them with the reference genome, analyze mRNA sequence and abundance information, and analyze gene structure and new transcripts generated. All the above processes were performed by Novogene Technology. For the gene function analysis, we first selected genes that differ in expression between DMSO and NCT-treated groups, and then performed Heatmap analysis, KEGG pathway analysis with Novomagic online platform from Novogene.

Chromatin immunoprecipitation (CHIP) assay

Astrocytes were pre-treated with DMSO or NCT for 2 h and then were treated with 0.1 μg/mL LPS for 4 h, and ChIP assay was performed based on the manufacturer's instructions (Beyotime Biotechnology P2080S). In briefly, astrocytes were cross-linked on ice containing formaldehyde for 30 min and sonicated to obtain DNA fragments with an average length of 500 to 1000 bp. The fragment lysates were immunoprecipitated with specific antibodies (IgG, Acetyl-Histone H3-K9, Acetyl-Histone H3-K27) and

slowly rotated overnight at 4°C. On the next day, protein A/G magnetic beads/salmon sperm DNA beads were added and rotated at 4°C for 60 min to precipitate protein-DNA complexes identified by primary antibodies. After the washing steps, the elution supernatant was treated with 5 M NaCl and heated at 45°C for 1 h to release the cross-linking between proteins and genomic DNA. On the third day, genomic DNA extracted by phenol and chloroform methods was used as templates for real-time PCR. The data were normalized by the input DNA of each sample. The primers used for qPCR were listed below: IL-6 Pro FP: 5’-TCCCATCAAGACATGCTCAA-3’, IL-6 Pro RP: 5’-AGGAAGGGGAAAGTGTGCTT-3’; IL-1β Pro FP: 5’-GGGAGAAGCTTGATGG G A AT-3’, IL-1β Pro RP: 5’-TATCTGCCACCCCTTGAC TT-3’.

NAD+/NADH abundance measurement

Astrocytes cells were treated with DMSO or NCT (20 μm) for 2 h and then were treated with 0.1 μg/mL LPS for 4 h. After that, NAD^+ and NADH abundance was detected according to the manufacturer’s instructions of NAD^+/NADH kit (Beyotime S0175).

Supplementary Figure Legends

Supplementary Figure 1. NCT-503 does not reduce astrocyte viability.

(A) Astrocyte viability at 0 h and 4 h after treatment with NCT-503 or DMSO followed by LPS stimulation, n=9. The data are means ± S.D. ***P < 0.001 by Two-Way ANOVA analysis followed by Bonferroni Test.

Supplementary Figure 2. PHGDH inhibition regulates histone acetylation in a SIRT-independent manner.

(A) Representative immunoblot analysis of H3K9Ac and H3K27Ac in siNC or siPHGDH-transfected astrocytes after LPS stimulation for 0 h, 2 h, and 4 h. (B) Representative immunoblot analysis of H3K9Ac and H3K27Ac in DMSO or NCT-503-treated astrocytes after LPS stimulation for 0 h, 2 h, and 4 h. (C) Representative immunoblot analysis of H3K9Ac and H3K27Ac in astrocytes transfected with siNC or siPHGDH and treated with SIRT inhibitor EX527 or 3-TYP for 2 h, and stimulated with LPS stimulation for 4 h. The loading controls were duplicated when testing H3K9Ac and H3K27Ac. (D)qRT-PCR analysis of TNFA, IL-1b, and IL-6 mRNA expression in DMSO or NCT-503-treated astrocytes followed by treatment with EX527 and 3-TYP for 2 h and LPS stimulation for 4 h, n=3. The data are means ± S.D., for all panels: *P < 0.05, **P <0.01, n.s., no significance by Two-Way ANOVA analysis followed by Bonferroni Test (A) and One-Way ANOVA analysis followed by Tukey’s Multiple Comparison Test (D).

Supplementary Figure 3. Schematic of experimental timeline for in vivo experiments.

(A) Schematic of experimental timeline for Figure 7.

Figure S1

A

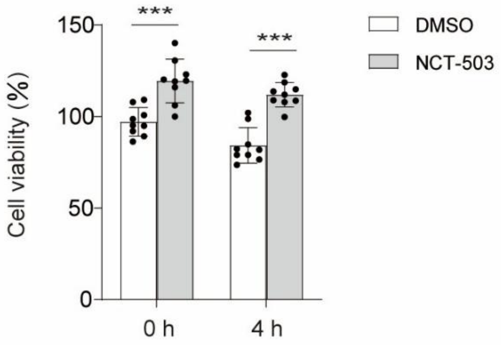

Figure S2

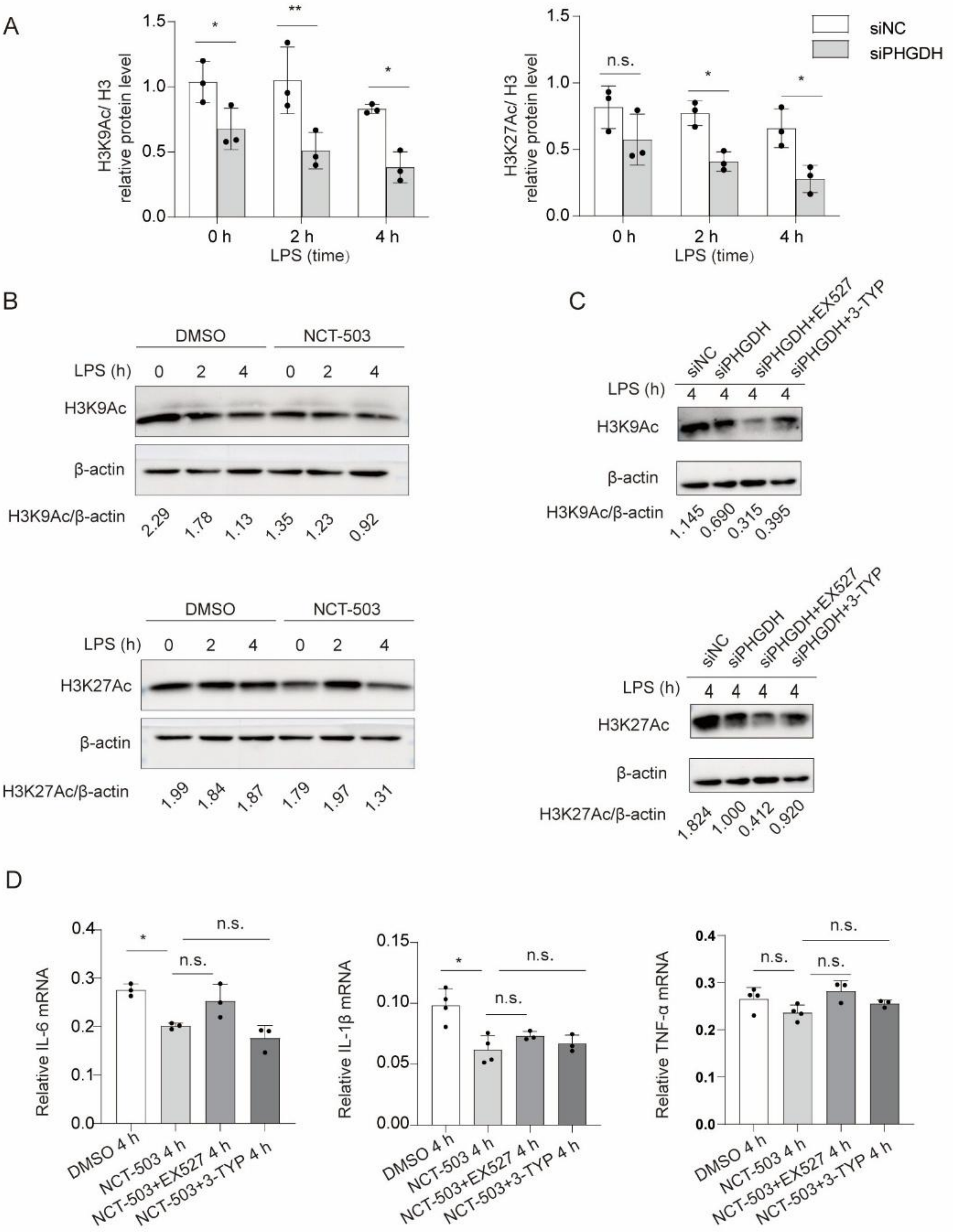
A
H3K9Ac/ H3 relative protein level
LPS (time)
0 h
2 h
4 h
H3K27Ac/ H3 relative protein level
siNC
siPHGDH
n.s.
B
DMSO
NCT-503
LPS (h)
H3K9Ac
β-actin
H3K9Ac/β-actin
2.29 1.78 1.13 1.35 1.23 0.92
H3K27Ac
H3K27Ac/β-actin
1.99 1.84 1.87 1.79 1.97 1.31
C
siNC
siPHGDH
siPHGDH+EX527
siPHGDH+3-TYP
H3K9Ac/β-actin
1.145 0.690 0.315 0.395
H3K27Ac/β-actin
1.824 1.000 0.412 0.920
D
Relative IL-6 mRNA
Relative IL-1β mRNA
Relative TNF-α mRNA
DMSO 4 h
NCT-503 4 h
NCT-503+EX527 4 h
NCT-503+3-TYP 4 h

Figure S3

A

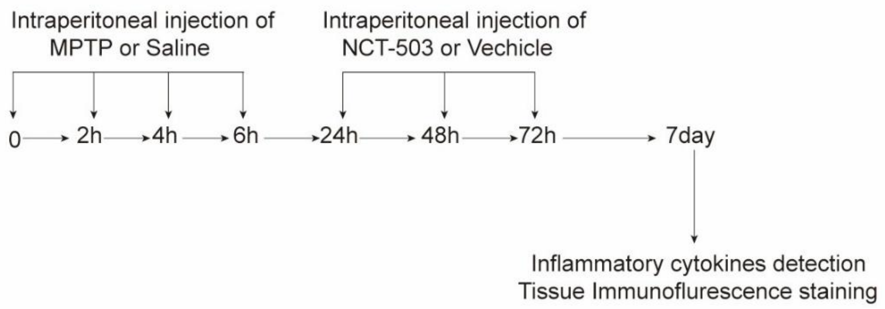
Intraperitoneal injection of MPTP or Saline
Intraperitoneal injection of NCT-503 or Vechicle
0
2h
4h
6h
24h
48h
72h
7day
Inflammatory cytokines detection
Tissue Immunoflurescence staining
