# Supplementary figures and images for: PHGDH-mediated serine synthesis in astrocytes supports neuroinflammation by sustaining NADH level to promote histone acetylation
Source: Cell Death Dis. 2025 May 18;16(1):397. doi: 10.1038/s41419-025-07732-8 (PMC12086227; doi:10.1038/s41419-025-07732-8)

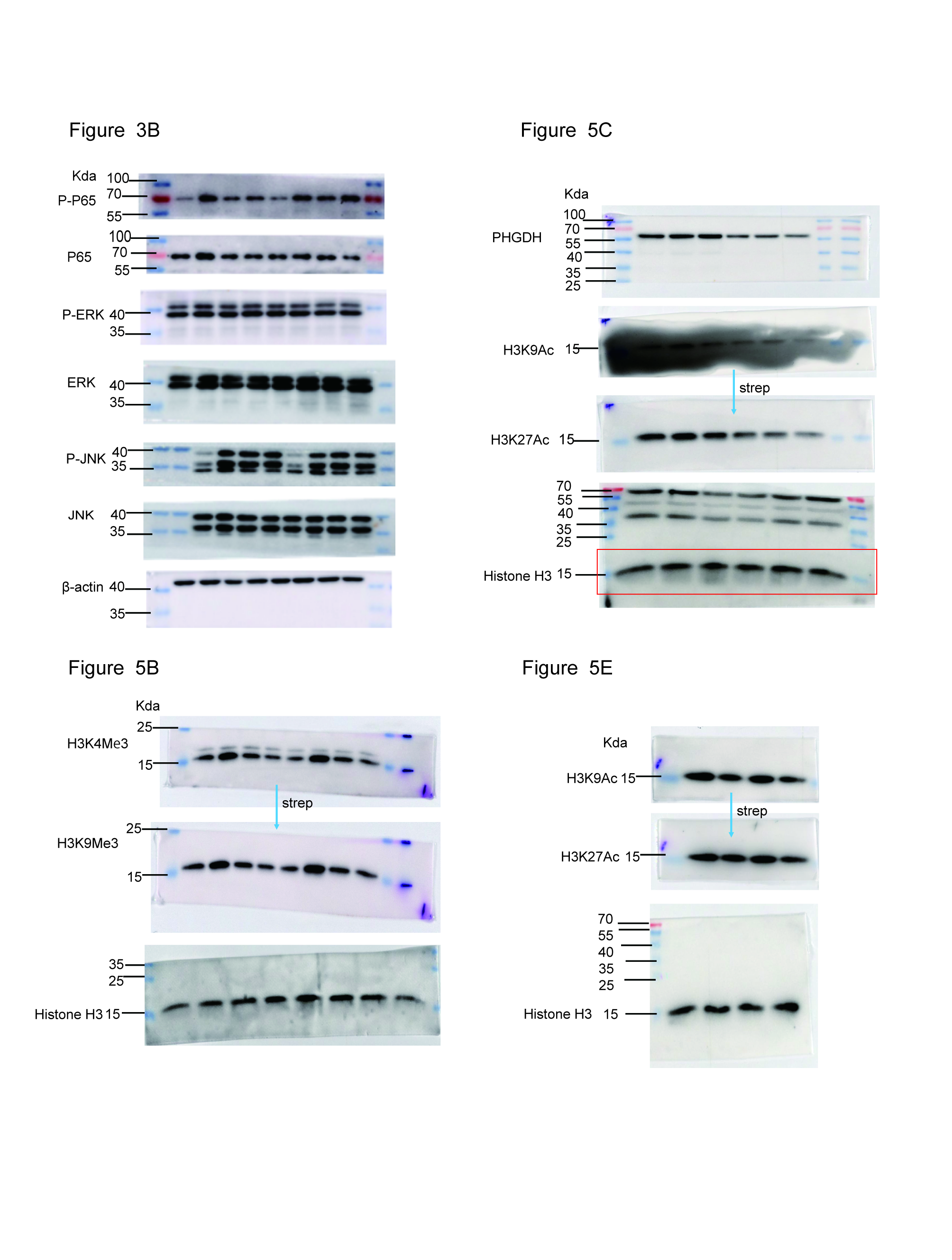

Supplement: Supplementary file 2 — Supplementary WB RAW data [file 41419_2025_7732_MOESM2_ESM.tif]
